# Supplementary material for: Bayesian inference for spatio-temporal stochastic transmission of plant disease in the presence of roguing: A case study to characterise the dispersal of Flavescence dorée
Source: PLoS Comput Biol. 2023 Sep 1;19(9):e1011399. doi: 10.1371/journal.pcbi.1011399 (PMC10501664; doi:10.1371/journal.pcbi.1011399)
Supplement: S1 Text — A: Equations used to model the infection pressure. B: Description of the MCMC algorithm. C: Simulation algorithm. (PDF) [file pcbi.1011399.s001.pdf]

Bayesian inference for spatio-temporal stochastic transmission of plant disease in the presence of roguing: a case study to characterise the dispersal of Flavescence dorée

Hola Kwame Adrakey, Gavin J. Gibson, Sandrine Eveillard, Sylvie Malembic-Maher and Frederic Fabre

Supplementary Text S1

Text A: Equations used to model the infection pressure

Four equations were used to model the infection pressure $\phi_i(t)$ exerted on host i at year t. They correspond to the combination of two hypotheses regarding the external source $\epsilon(t)$ (1: constant between years, or 2: variable between years) with two hypotheses regarding a cultivar infectivity effect (1: presence, by setting $c_j = 1.42$ for Cabernet-Sauvignon source plant and $c_j = 1$ for Merlot source plant or 2: absence by setting $c_j = 1$ regardless of the cultivar of the source plant).

$$\phi_i(t) = \epsilon + \beta \sum_{j\in I_{t-1}} K(r_{ij};\alpha)\mathbb{1}_{j\in F_1} + \beta \sum_{j\in I_{t-1}} K(r_{ij};\alpha)\mathbb{1}_{j\in F_2\cup F_3} \quad (1)$$

$$\phi_i(t) = \epsilon(t) + \beta \sum_{j\in I_{t-1}} K(r_{ij};\alpha)\mathbb{1}_{j\in F_1} + \beta \sum_{j\in I_{t-1}} K(r_{ij};\alpha)\mathbb{1}_{j\in F_2\cup F_3} \quad (2)$$

$$\phi_i(t) = \epsilon + \beta \sum_{j\in I_{t-1}} c_j K(r_{ij};\alpha)\mathbb{1}_{j\in F_1} + \beta \sum_{j\in I_{t-1}} c_j K(r_{ij};\alpha)\mathbb{1}_{j\in F_2\cup F_3} \quad (3)$$

$$\phi_i(t) = \epsilon(t) + \beta \sum_{j\in I_{t-1}} c_j K(r_{ij};\alpha)\mathbb{1}_{j\in F_1} + \beta \sum_{j\in I_{t-1}} c_j K(r_{ij};\alpha)\mathbb{1}_{j\in F_2\cup F_3} \quad (4)$$

Text B: Description of the MCMC algorithm

We employ the MCMC scheme, especially Metropolis within Gibbs sampling [1–7]. We assume vague exponential priors for all the parameters and derive the posterior distribution of the probability of removing a plant from reasons other than FD q and the joint posterior distribution of the other parameters respectively as:

$$q \sim Beta\left(1 + \sum_{j\in\mathcal{R}} \mathbb{1}_{\{r_j=0\}}, 1 + \sum_{j\in\mathcal{R}} (t_j^{\mathcal{R}} - 1) + t_{max}|\mathcal{R}| + \sum_{j\in\mathcal{R}} \mathbb{1}_{\{r_j=1\}}\right) \tag{5}$$

$$\begin{aligned} P(\alpha,\epsilon,\beta|y) = \quad & \textstyle\prod_{j\in\mathcal{R}} \Big[\prod_{i=1}^{t_j^{\mathcal{R}}-1} \exp\left(-\phi_j(i)\right) \left[\left(1-\exp\left(-\phi_j(t_j^{\mathcal{R}})\right)\right) \mathbb{1}_{r_j=1} + \right. \\ & \left. \exp\left(-\phi_j(t_j^{\mathcal{R}})\right) \mathbb{1}_{\{r_j=0\}}\right]\Big] \times \\ & \textstyle\prod_{j\in\bar{\mathcal{R}}} \prod_{i=1}^{t_{max}} \exp\left(-\phi_j(i)\right) \end{aligned} \tag{6}$$

Algorithm to generate samples from the posterior distribution using Metropolis within Gibbs Sampling

1. Initiate the chain with values β^0, α^0, ϵ^0, q^0, $\underline{t}^{\mathcal{R}_u^0}$ and $\mathcal{R}_u^0$.
2. Update q using Gibbs Sampling by drawing from it corresponding full conditional distribution.
3. Update to α, ϵ and β is performed using Metropolis-Hastings
   (a) Accept the new parameter θ_i^{new} with probability

$$p_{acc} = \min\left\{1, \frac{p(\theta_{\mathbf{i}}{}^{new}|\theta_{-i},\mathbf{y})}{p(\theta_{\mathbf{i}}{}^{old}|\theta_{-i},\mathbf{y})}\right\} \tag{7}$$

   where $\theta = (\alpha,\epsilon,\beta)$ and the index $i = 1,2,3$ specifies the position of the corresponding parameter. θ_{-i} represents the vector of parameters exclude the parameter at position i.

4. Update the reason an individual was removed before 2018 using reversible-jump mcmc:

   Repeat

   (a) Select a host $j \in \mathcal{R}_u$, a host removed before 2018.
   (b) If j was removed due to FD infection, propose to do one of the following:
      i. Move its removal date with probability 1/2. We do this by proposing a new $t_j^{\mathcal{R}^{new}}$ and accept it with probability

$$p_{acc} = \min\left\{1, \frac{L(\alpha,\beta,\epsilon,q;\underline{t}^{\mathcal{R}_u^{new}}\mathcal{R}_u^{new})}{L(\alpha,\beta,\epsilon,q,\underline{t}^{\mathcal{R}_u^{old}}\mathcal{R}_u^{old}))}\right\} \tag{8}$$

      ii. Change the reason for removal to be another reason other than infection ($r_j = 0$) with probability 1/2 i.e. the individual remains susceptible at the time of removal. We accept the new set of reasons with the probability

$$p_{acc} = \min\left\{1, 2\frac{L(\alpha,\beta,\epsilon,q;\underline{t}^{\mathcal{R}_u}\mathcal{R}_u^{new})}{L(\alpha,\beta,\epsilon,q,\underline{t}^{\mathcal{R}_u}\mathcal{R}_u^{old}))}\right\} \tag{9}$$

(c) If j was removed because of reasons other than an infection, we propose to change the reason to an FD infection. We accept the new set of reasons with the probability

$$p_{acc} = p_{acc} = \min\left\{1, \frac{1}{2}\frac{L(\alpha, \beta, \epsilon, q; \underline{t}^{\mathcal{R}_u}\mathcal{R}_u^{new})}{L(\alpha, \beta, \epsilon, q, \underline{t}^{\mathcal{R}_u}\mathcal{R}_u^{old}))}\right\} \tag{10}$$

5. Repeat i)-iv) until convergence.

References

1. Gibson GJ, Renshaw E. Estimating parameters in stochastic compartemental model using Markov chain methods. IMA J Math Appl Med. 1998;15:19–40.

2. Neri F, Cook F, Gibson G, Gottwald T, Galligan C. Bayesian analysis for inference of an emerging epidemic: citrus canker in urban landscapes. PLoS Comput Biol. 2014;10.

3. Parry M, Gibson GJ, Parnell S, Gottwald TR, Irey MS, Gast TC, et al. Bayesian inference for an emerging arboreal epidemic in the presence of control. PNAS. 2014;111(17):6258–6262. doi:10.1073/pnas.1310997111.

4. Lau M, Marion G, Streftaris G, Gibson G. A Systematic Bayesian Integration of Epidemiological and Genetic Data. PLoS Comput Biol. 2015;11. doi:doi:10.1371/journal.pcbi.1004633.

5. MacCalman L, McKendrick IJ, Denwood M, Gibson G, Catterall S, Innocent G, et al. MAPRA: modelling animal pathogens: review and adaptation. EFSA Supporting Publications. 2016;13(12):1112E. doi:10.2903/sp.efsa.2016.EN-1112.

6. Lau M, Gibson G, Adrakey H, McClelland A, Riley S, Zelner J, et al. A mechanistic spatio-temporal framework for modelling individual-to-individual transmission—With an application to the 2014-2015 west Africa Ebola outbreak. PLoS Comput Biol. 2017;13:e1005798. doi:doi:10.1371/journal.pcbi.1005798.

7. Adrakey H, Streftaris G, Cuniffe N, Gottwald T, Gilligan C, Gibson G. Evidence-based controls for epidemics using spatio-temporal stochastic models in a Bayesian framework. J R Soc Interface. 2017;14.

Text C: Simulation algorithm

FD simulation algorithm

1. Initialise $t = t_0$, the initial time, and set the location of the initial infection to be $X_0 = (x_0, y_0)$, the number of infectious hosts $n = 1$.

2. New infections (or removals) are generated as follows. Suppose that at time t there have been k active infections and let $P_j(t+1)$ the probability that a susceptible plant j gets infected at year $t+1$. Recall from the main text that

$$P_j(t) = 1 - exp\left(-\phi_j(t)\right). \tag{11}$$

where

$$\phi_j(t) = \beta \sum_{i \in I_{t-1}} K(x, y; \alpha) \tag{12}$$

Draw $u_1 \sim U(0,1)$ and $u_2 \sim U(0,1)$

3. If $u_1 < P_j(t+1)$, then the plant at position x is infected else it remains susceptible and set $t = t+1$.

4. If $u_2 < \tau$ for any of the k active infected plants, remove such a plant and replace it with a new healthy plant and set $t = t+1$. Recall that τ represents the efficiency of the removal.

5. Repeat $2 - 4$ until a stopping criterion is reached (e.g. $t \geq T$).
