# Supplementary material for: Bayesian inference for spatio-temporal stochastic transmission of plant disease in the presence of roguing: A case study to characterise the dispersal of Flavescence dorée
Source: PLoS Comput Biol. 2023 Sep 1;19(9):e1011399. doi: 10.1371/journal.pcbi.1011399 (PMC10501664; doi:10.1371/journal.pcbi.1011399)
Supplement: S2 Text — (PDF) [file pcbi.1011399.s002.pdf]

Bayesian inference for spatio-temporal stochastic transmission of plant disease in the presence of roguing: a case study to characterise the dispersal of Flavescence dorée

Hola Kwame Adrakey, Gavin J. Gibson, Sandrine Eveillard, Sylvie Malembic-Maher and Frederic Fabre

Supplementary Text S2

<u>Parameter's estimates for the 20 models fitted</u>

For the 20 models fitted, the following parameters were estimated : (i) α, the scale parameter of the dispersal kernel, (ii) ϵ, the primary infection rate, (iii) β, the secondary infection rate, (iv) q, the rate of removal for reasons other than FD and (v) t_0, the year of first infection of fields $F_2 \cup F_3$. The estimated values of t_0 are given in Fig A and in Table A for all the other parameters.

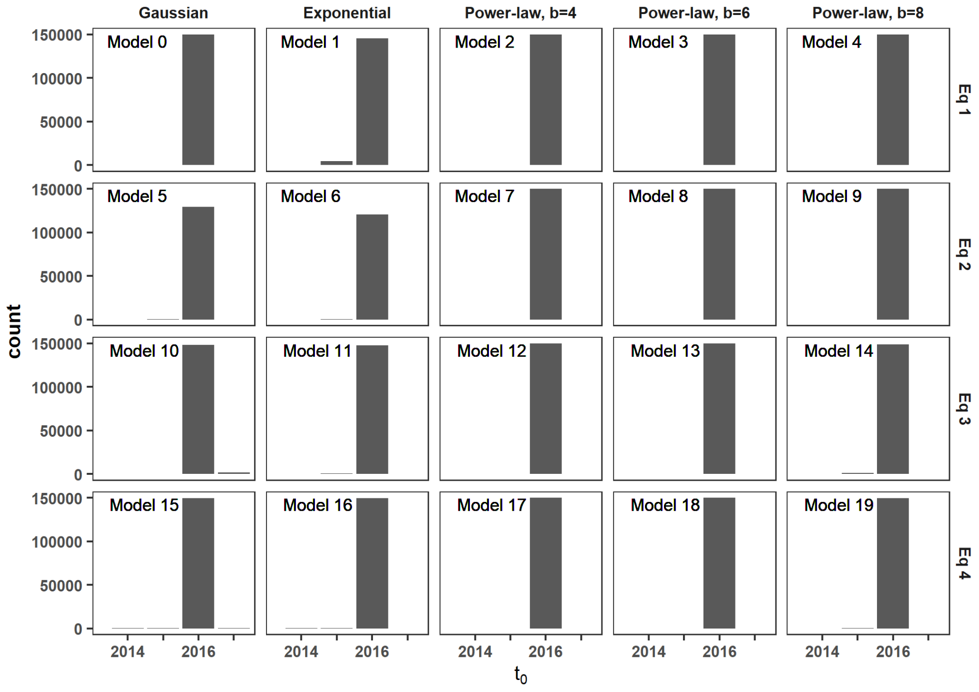

Fig A. Posterior distribution of the starting year of the epidemic in fields F_2 and F_3 according to the 20 models fitted. The 20 models differ according to their dispersal kernel (in column) and formulation of the infection pressure (in row).

Table A. Posterior mean, median and 95% credible region for the model parameters .

Parameter	0.25%	mean	median	97.5%	0.25%	mean	median	97.5%
		**Model0**				**Model1**		
α	54.440	60.067	59.897	66.325	47.180	55.388	55.146	65.019
β	5.439	5.905	5.902	6.381	3.132	3.395	3.393	3.674
ϵ	0.001	0.002	0.002	0.003	0.001	0.001	0.001	0.002
q	0.004	0.004	0.004	0.005	0.004	0.004	0.004	0.005
		**Model2**				**Model3**		
α	22.085	26.293	26.168	31.213	24.223	28.384	28.196	33.532
β	31.078	34.447	34.388	38.021	103.737	116.690	116.549	130.040
ϵ	0.001	0.001	0.001	0.002	0.001	0.002	0.001	0.002
q	0.004	0.004	0.004	0.005	0.004	0.004	0.004	0.005
		**Model4**				**Model5**		
α	25.763	30.305	29.960	37.149	53.366	58.839	58.705	65.239
β	211.501	242.079	241.945	271.588	5.349	5.830	5.827	6.329
ϵ	0.001	0.002	0.002	0.003	0.0001	0.0001	0.0001	0.0002
q	0.004	0.004	0.004	0.005	0.004	0.004	0.004	0.005
		**Model6**				**Model7**		
α	45.260	54.474	54.309	64.295	21.960	26.020	25.786	31.356
β	3.097	3.363	3.360	3.649	30.744	34.161	34.155	37.622
ϵ	0.0001	0.0001	0.0001	0.0002	0.0001	0.0001	0.0001	0.0002
q	0.004	0.004	0.004	0.005	0.004	0.004	0.004	0.005
		**Model8**				**Model9**		
α	23.880	27.835	27.626	33.026	24.760	28.789	28.558	34.256
β	103.672	116.340	116.275	129.163	216.128	243.486	243.630	270.947
ϵ	0.0001	0.0001	0.0001	0.0002	0.0001	0.0001	0.0001	0.0002
q	0.004	0.004	0.004	0.005	0.004	0.004	0.004	0.005
		**Model10**				**Model11**		
α	57.275	63.030	62.841	69.966	52.261	60.949	60.608	71.257
β	3.902	4.235	4.232	4.585	2.266	2.458	2.457	2.662
ϵ	0.001	0.003	0.002	0.004	0.001	0.002	0.002	0.003
q	0.004	0.004	0.004	0.005	0.004	0.004	0.004	0.005
		**Model12**				**Model13**		
α	18.600	21.939	21.805	26.112	20.789	23.872	23.751	27.748
β	27.025	30.333	30.282	33.865	94.018	106.191	106.121	118.840
ϵ	0.001	0.002	0.002	0.003	0.001	0.002	0.002	0.003
q	0.004	0.004	0.004	0.005	0.004	0.004	0.004	0.005
		**Model14**				**Model15**		
α	80.149	101.963	101.039	127.108	55.882	61.305	61.122	67.819
β	103.701	112.734	112.521	123.013	3.796	4.152	4.152	4.510
ϵ	0.001	0.002	0.002	0.003	0.000	0.000	0.000	0.000
q	0.004	0.004	0.004	0.005	0.004	0.004	0.004	0.005
		**Model16**				**Model17**		
α	50.114	59.120	58.757	69.894	18.437	21.684	21.567	25.547
β	2.223	2.427	2.426	2.635	26.885	29.936	29.914	33.118
ϵ	0.0001	0.0001	0.0001	0.0002	0.0001	0.0001	0.0001	0.0002
q	0.004	0.004	0.004	0.005	0.004	0.004	0.004	0.005
		**Model18**				**Model19**		
α	20.074	22.953	22.848	26.518	75.503	98.206	98.088	122.133
β	94.462	106.099	105.959	118.383	101.944	111.427	111.334	121.829
ϵ	0.0001	0.0001	0.0001	0.0002	0.0001	0.0001	0.0001	0.0002
q	0.004	0.004	0.004	0.005	0.004	0.004	0.004	0.005
