# Supplementary material for: Bayesian inference for spatio-temporal stochastic transmission of plant disease in the presence of roguing: A case study to characterise the dispersal of Flavescence dorée
Source: PLoS Comput Biol. 2023 Sep 1;19(9):e1011399. doi: 10.1371/journal.pcbi.1011399 (PMC10501664; doi:10.1371/journal.pcbi.1011399)
Supplement: S1 Fig — (PDF) [file pcbi.1011399.s003.pdf]

Bayesian inference for spatio-temporal stochastic transmission of plant disease in the presence of roguing: a case study to characterise the dispersal of Flavescence dorée

Hola Kwame Adrakey, Gavin J. Gibson, Sandrine Eveillard, Sylvie Malembic-Maher and Frederic Fabre

Supplementary Figure S1

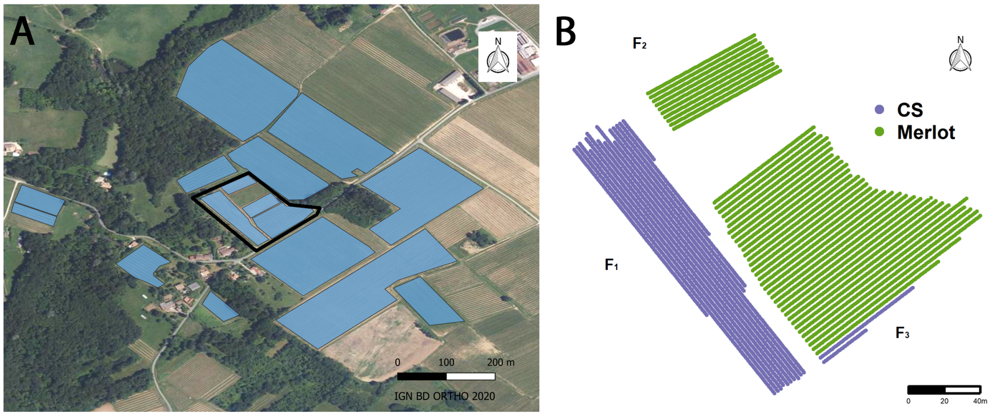

Fig S1. Map of the three vineyard fields F_1, F_2 and F_3 in their landscape. A: Map of the study area in Faleyras, a district in South West France. The map shows the polygons of targeted fields F_1, F_2 and F_3 (highlighted in the box) and the neighbouring fields within 300 m. B: Initial state of the fields where 2259 Cabernet Sauvignon (CS) were planted in field F_1, 677 Merlot in field F_2, and 3025 Merlot and 95 Cabernet Sauvignon in field F_3. The above map (panel A) is created using the package leaflet in R software. The basemap is from IGN BD Ortho IGN BD Ortho tiles, a map published under the license Etalab2.
