# Supplementary material for: Bayesian inference for spatio-temporal stochastic transmission of plant disease in the presence of roguing: A case study to characterise the dispersal of Flavescence dorée
Source: PLoS Comput Biol. 2023 Sep 1;19(9):e1011399. doi: 10.1371/journal.pcbi.1011399 (PMC10501664; doi:10.1371/journal.pcbi.1011399)
Supplement: S2 Fig — (PDF) [file pcbi.1011399.s004.pdf]

Bayesian inference for spatio-temporal stochastic transmission of plant disease in the presence of roguing: a case study to characterise the dispersal of Flavescence dorée

Hola Kwame Adrakey, Gavin J. Gibson, Sandrine Eveillard, Sylvie Malembic-Maher and Frederic Fabre

Supplementary Figure S2

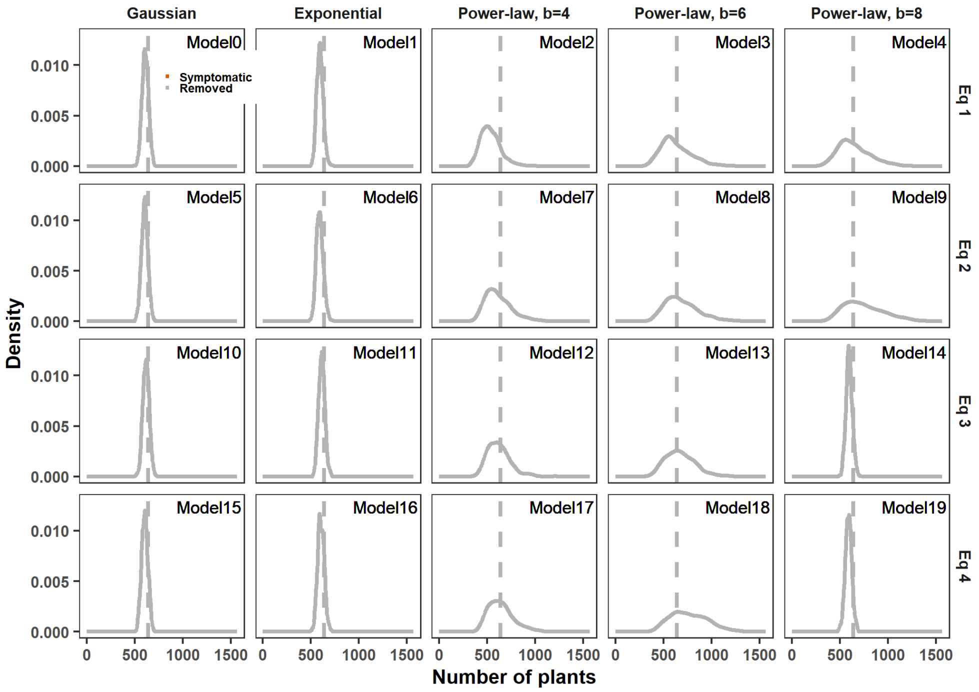

Fig S2. Comparison of the 20 models using the counts of observed symptomatic and removed plants in 2019. In each panel, corresponding to a model, the dotted line is the actual observed count and the density of counts are obtained from 1 000 simulations. The colors correspond to the symptomatic (red) and the removed (blue) plants. The 20 models differ according to their dispersal kernel (in column) and formulation of the infection pressure (in row). Symptomatic and removed plants in 2019 coincident here since we did not allow for removals for reasons other than FD in 2019 as this information was not available. In fact, this should have been available on the 2020 snapshot.
