# Supplementary material for: Bayesian inference for spatio-temporal stochastic transmission of plant disease in the presence of roguing: A case study to characterise the dispersal of Flavescence dorée
Source: PLoS Comput Biol. 2023 Sep 1;19(9):e1011399. doi: 10.1371/journal.pcbi.1011399 (PMC10501664; doi:10.1371/journal.pcbi.1011399)
Supplement: S3 Fig — (PDF) [file pcbi.1011399.s005.pdf]

Bayesian inference for spatio-temporal stochastic transmission of plant disease in the presence of roguing: a case study to characterise the dispersal of Flavescence dorée

Hola Kwame Adrakey, Gavin J. Gibson, Sandrine Eveillard, Sylvie Malembic-Maher and Frederic Fabre

Supplementary Figure S3

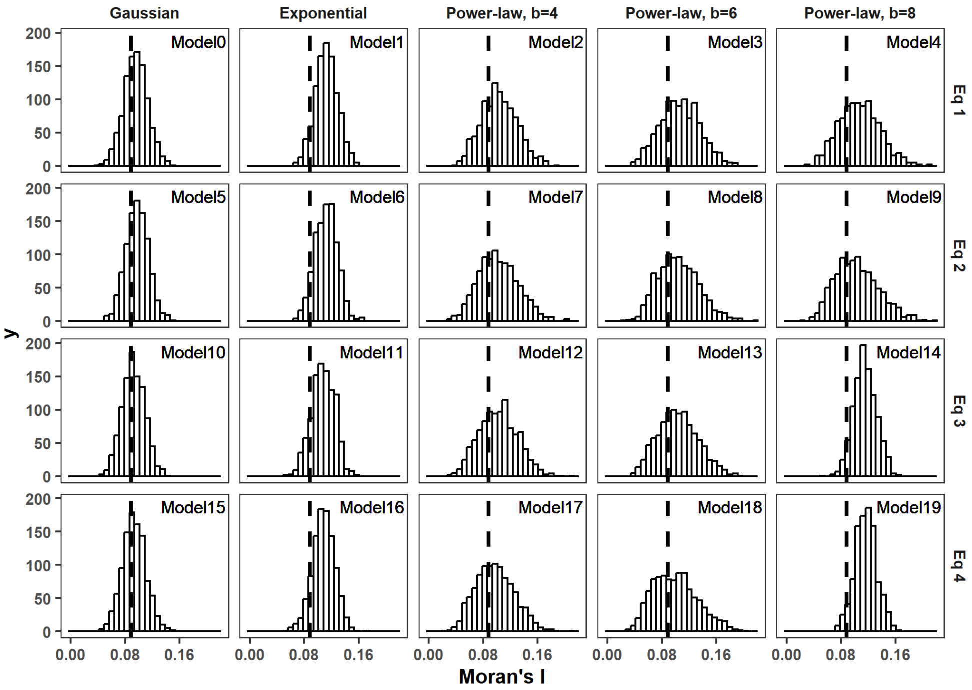

Fig S3. Comparison of the 20 models using the spatial correlation index of Moran. Posterior predictive distributions of spatial correlation using Moran's I index [1]. In each panel, corresponding to a model, the black line represents the actual measure of the observed data. The 20 models differ according to their dispersal kernel (in column) and formulation of the infection pressure (in row).

References

1. Getis A. Spatial interaction and spatial autocorrelation: a cross-product approach. Environ Plann A: Econ Space. 1977;23(9):1269—1277.
